# Impact of flanking chromosomal sequences on localization and silencing by the human non-coding RNA XIST

**DOI:** 10.1186/s13059-015-0774-2

**Published:** 2015-10-02

**Authors:** Angela D. Kelsey, Christine Yang, Danny Leung, Jakub Minks, Thomas Dixon-McDougall, Sarah E.L. Baldry, Aaron B. Bogutz, Louis Lefebvre, Carolyn J. Brown

**Affiliations:** Department of Medical Genetics, Molecular Epigenetics Group, Life Sciences Institute, University of British Columbia, Vancouver, Canada; Ludwig Institute for Cancer Research, University of California at San Diego School of Medicine, La Jolla, CA USA; Division of Life Science, The Hong Kong University of Science and Technology, Clear Water Bay, Hong Kong, China

**Keywords:** XIST, Long non-coding RNA, Dosage compensation, X-chromosome inactivation, Nucleolar localization, Facultative heterochromatin, SMCHD1, macroH2A, H3K27me3, H4K20me1

## Abstract

**Background:**

X-chromosome inactivation is a striking example of epigenetic silencing in which expression of the long non-coding RNA XIST initiates the heterochromatinization and silencing of one of the pair of X chromosomes in mammalian females. To understand how the RNA can establish silencing across millions of basepairs of DNA we have modelled the process by inducing expression of *XIST* from nine different locations in human HT1080 cells.

**Results:**

Localization of XIST, depletion of Cot-1 RNA, perinuclear localization, and ubiquitination of H2A occurs at all sites examined, while recruitment of H3K9me3 was not observed. Recruitment of the heterochromatic features SMCHD1, macroH2A, H3K27me3, and H4K20me1 occurs independently of each other in an integration site-dependent manner. Silencing of flanking reporter genes occurs at all sites, but the spread of silencing to flanking endogenous human genes is variable in extent of silencing as well as extent of spread, with silencing able to skip regions. The spread of H3K27me3 and loss of H3K27ac correlates with the pre-existing levels of the modifications, and overall the extent of silencing correlates with the ability to recruit additional heterochromatic features.

**Conclusions:**

The non-coding RNA XIST functions as a *cis*-acting silencer when expressed from nine different locations throughout the genome. A hierarchy among the features of heterochromatin reveals the importance of interaction with the local chromatin neighborhood for optimal spread of silencing, as well as the independent yet cooperative nature of the establishment of heterochromatin by the non-coding XIST RNA.

**Electronic supplementary material:**

The online version of this article (doi:10.1186/s13059-015-0774-2) contains supplementary material, which is available to authorized users.

## Background

To avoid a functional gene dosage imbalance between the sexes, one of the two X chromosomes in female placental mammals is transcriptionally silenced [1]. This process of X-chromosome inactivation (XCI) occurs early in development and is generally random in all human tissues with either the paternal or maternal X chromosome becoming the inactive X (Xi). The X-inactivation centre (*XIC*), which is located at Xq13 in humans, is the region of the X that is necessary for the chromosome to be inactivated, and contains the *XIST* gene (X-inactive specific transcript) [2–4]. Remarkably, the approximately 17 kb spliced and polyadenylated long non-coding XIST RNA uniquely localizes to the chromosome from which it is transcribed [5]. The coating of the Xi by the XIST RNA results in a substantial epigenetic transformation, losing epigenetic modifications associated with active chromatin (notably histone acetylation) and gaining modifications associated with inactive chromatin (including H3K27me3, H3K9me2/3, H4K20me1, and H2AK119u1). The Xi also becomes enriched in several other proteins, including the histone variant macrohistone H2A (macroH2A), the nuclear matrix protein hnRNPU, and the epigenetic regulators SMCHD1 and ASH2L ([6, 7], reviewed in [8]). In addition, the Xi is peripherally or perinucleolarly localized [9] with perinucleolar targeting during S phase suggested to be important for maintenance of silencing. A further feature of the Xi is silencing of repetitive elements, as visualized by loss of RNA hybridization with a Cot-1 probe for repetitive DNA [10] resulting in what has been termed a ‘Cot-1 hole’.

The timing of acquisition of these features has been best studied in mouse, where the differentiation of embryonic stem cells (ESCs) provides an *in vitro* model for the events of XCI (reviewed in [11]). Early studies in mouse suggested the presence of a developmental window beyond which Xist was unable to induce X-chromosome inactivation [12], although macroH2A could be recruited [13]. After this stage, *Xist* expression was no longer required for maintenance of silencing [14], consistent with studies in human showing maintenance of silencing in the absence of *XIST* [15, 16]. SATB1 has been suggested to be involved in defining such a window for *Xist* function [17]; however, *Satb1/Satb2*-deficient mice are able to undergo X inactivation [18]. More recent studies have shown that induction of *Xist* can recruit H3K27me3 in mouse somatic cells [19], and XIST induction recruits multiple features of the Xi in human somatic cells [10, 20]. In addition, an ongoing role for *Xist* in stable silencing of the Xi has been shown by loss of *Xist* resulting in gene reactivation, loss of perinucleolar association and loss of H3K27me3 [21]. Loss of X-linked gene silencing is enhanced by disruption of DNA methylation and other pathways that cooperate with Xist, thus reactivation of X-linked markers has been used to identify additional players in the pathway [22–24]. Characterization of the ongoing role for *XIST* in somatic cells has important implications for disease, as highlighted by a recent study showing that deletion of *Xist* results in hematological malignancies in female mice due to reactivation of X-linked genes [25].

There is substantial evidence for cooperativity of multiple silencing pathways in the initial silencing of the chromosome, with XCI able to proceed in the absence of key components of the silencing machinery such as PRC2 [26–28], PRC1 [29], or macroH2A [30]. In addition to multiple factors cooperating in the process of XCI, different X-linked genes may be silenced (or maintained silent) by different players. For example, mutation of the *Smchd1* gene results in loss of DNA methylation and partial to full reactivation of approximately 20 % of the X-linked genes in mouse [31, 32]. Surprisingly, many of the marks of an Xi can be recruited by a mouse transgene containing a deletion that makes the Xist RNA defective in silencing, although often the recruitment is not as effective as seen with a full-length Xist (reviewed in [8]). This silencing defective Xist RNA is also able to form a Cot-1 hole [33], consistent with the Cot-1 hole not reflecting X-linked gene silencing [34], but rather a core of silenced non-coding DNA [35].

Spread of silencing to autosomal genes has been observed in unbalanced X/autosome translocations; however, the extent of autosomal silencing is highly variable in both humans (reviewed in [36]) and mice [37]. Silencing of autosomal genes has also been observed upon integration of *Xist/XIST* transgenes into autosomes, and localization of the RNA to the autosome is able to induce many features of the Xi including nucleolar localization [10, 20, 21, 38]. Recently, an *XIST* transgene was integrated into chromosome 21 in induced pluripotent stem cells from an individual with Down’s syndrome and corrected gene expression from chromosome 21 to near normal disomic levels [39]. Together, these studies demonstrate the XIST RNA is able not only to spread along autosomal material but also to recruit some of the heterochromatic features associated with XCI to autosomes. The silencing of the trisomic chromosome 21 in induced pluripotent stem cells was proposed as a first step towards ‘chromosome therapy’ [39], and for such uses of *XIST* a better understanding of the influence of the chromatin neighborhood is necessary.

XIST expression is required to induce the cascade of changes that cooperatively silence the X, but relatively little is known about the process by which the non-coding RNA recruits these changes. In human somatic cells we have reported the recruitment of several features of XCI, including gene silencing, following *XIST* expression from an inducible transgene [20, 40]. The separation of *XIST* expression from the myriad of changes that occur during differentiation provides an opportunity to dissect the role of XIST in XCI. By using this inducible XIST transgene to examine the influence of XIST expression induced from nine different integration sites we aimed to establish a hierarchy to the features that are recruited by XIST and determine if any were influenced by the genomic context of the XIST integration. We were able to identify features (Cot-1 hole formation, perinucleolar localization, proximal reporter silencing, and H2AK119u1) that are recruited to all integrations and thus are compatible with any of the sites/genomic contexts tested, a feature that is not recruited to any site examined (H3K9me3) while another set of features (macroH2A, H3K27me3, SMCHD1, and H4K20me1 recruitment) are dependent on genomic context, but appear independent of each other.

## Results

### XIST RNA expression results in depletion of Cot-1 RNA and increased perinucleolar location

In the HT1080 cells an inducible *XIST* cDNA has been previously reported to localize in *cis* to the chromosome from which it is transcribed, resulting in the recruitment of some chromatin modifications and repression of both flanking reporter genes as well as flanking endogenous genes [20, 40]. To test whether there are differences in the functionality of the XIST RNA due to influences of local DNA sequences and chromatin environment we integrated a single copy of the full-length inducible *XIST* cDNA into nine different FRT sites in HT1080 cells containing a constitutive Tet-repressor transgene to allow induction of XIST by treatment of the cells with the tetracycline analog doxycycline (DOX). The sites of the FRT integrations were identified by inverse PCR and sequencing to be 1p, 3q, 4q, 7p, 7q, 8p, 12q, 15q, and an X chromosome FRT site at Xq23 was previously reported [41]. The integration sites (described in Additional file 1) are in both G-dark and G-light regions (four and five, respectively), and three integrations occurred within a gene.

**Fig. 1:**
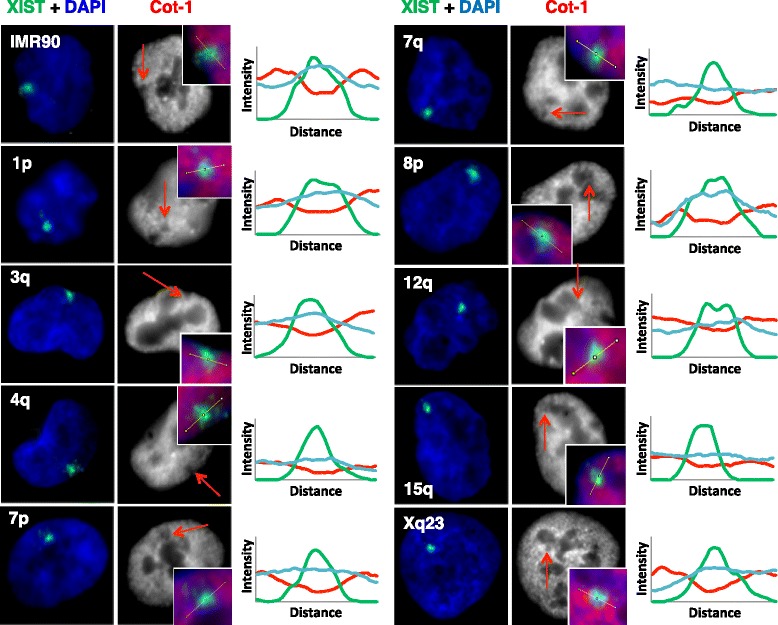
XIST RNA localizes and forms Cot-1 holes when expressed from nine different integration sites. Shown is XIST RNA FISH (green) upon expression of an inducible *XIST* transgene integrated into the indicated chromosomal locations in HT1080 cells. IMR90 cells (a female fibroblast line) are shown as a positive comparator. Cells were counter-stained with DAPI (blue) and co-hybridized with Cot-1 (labelled with spectrum-red, but shown in grayscale). Arrow indicates the location of the XIST signal and reduction in Cot-1 staining. Graphs to the right show the RGB intensities across the lines shown in the picture inserts drawn through the XIST clouds (XIST (green), Cot-1 (red), and DAPI (blue))

For each clone, the inducible promoter was activated for 5 days and the localization of XIST RNA assessed by RNA FISH. At each integration site the XIST RNA was able to localize and formed an XIST cloud comparable to that observed in normal female cells (Fig. 1). The level of XIST RNA varied between cell lines and also within different cultures of the same cell line, showing from five- to 30-fold induction of XIST after 5 days of DOX (Additional file 1). The integrations into 3q, 7p, and 15q (all G-dark) showed lower expression by q-RT-PCR, which is consistent with a significantly smaller signal for XIST for the 3q integration clone relative to all other integrations except for 15q (*P* ≤0.01). Co-hybridization with fluorescently labelled Cot-1 also showed depletion of Cot-1 hybridization coincident with the XIST cloud at each integration site, observable in the line diagrams of signal intensity across the XIST cloud (Fig. 1). Recently stable Cot-1 repeat RNA has been shown to be associated with euchromatic chromosomes, yet excluded from the Xi resulting in the Cot-1 RNA hole [42]. We noted differences in the intensity of the Cot-1 holes; however, in attempting to quantify such differences it became apparent that the XIST RNA signal was often at the nuclear or nucleolar periphery in the HT1080 cells, and that measuring the intensity of the Cot-1 hole could be influenced by nuclear location. The Xi is generally located at the nuclear or nucleolar periphery; however, as these were autosomal integration sites, it seemed that expression of XIST might be altering nuclear location.

To test whether XIST expression was relocating the chromosome from which it was expressed, the XIST signal was scored for being in contact with either the nuclear periphery or the nucleolus, prior to and after XIST induction. Prior to DOX induction there was only a small focus of XIST expression (see [20]); however this signal was sufficient to identify the location of the integrated *XIST*. In six of the nine integration sites, induction with DOX resulted in a significant increase in perinucleolar localization (*P* ≤0.05), a trend shared with the other integration sites (Table 1). Perinuclear association, on the other hand, showed no significant difference for five of the integrations, with three integrations showing a significant increase and the 8p integration showing a significant decrease (*P* ≤0.01; Table 1). The full distribution of localization before and after induction of XIST is shown in Additional file 2. The 8p integration site showed the highest proportion of perinucleolar-associated XIST signals (56 %) and also the greatest increase in perinucleolar association following XIST induction (27 %). In mouse, localization of Xist to the perinucleolar compartment was shown to be necessary for the silencing activity of Xist [21], leading us to question whether the differing nucleolar recruitment and Cot-1 hole formation that we observed might be reflective of silencing ability.

**Table 1:** Increased nucleolar association of chromosomes expressing transgenic XIST

Integration site	% perinuclear	% perinuclear	% perinuclear	% perinucleolar	% perinucleolar	% perinucleolar
	No DOX	DOX	CHANGE	No DOX	DOX	CHANGE
1p	27	34	7	10	36	16^a^
3q	78	70	−8	10	18	8
4q	57	74	17^b^	12	25	14^a^
7p	32	27	−5	29	47	18^a^
7q	30	53	23^a^	28	39	11^c^
8p	59	39	−21^a^	29	56	27^a^
12q	36	39	3	18	35	17^a^
15q	24	36	14^c^	46	53	7
Xq23	42	46	5	31	41	10

The numbers shown are based on three independent experiments for the DOX results and one experiment for the No DOX results, with ≥50 cells counted for each integration site in each experiment. Chi-squared test (^a^ *P* ≤0.001; ^b^ *P* ≤0.01; ^c^ *P* ≤0.05)

### XIST RNA expression silences nearby reporter and endogenous genes

**Fig. 2:**
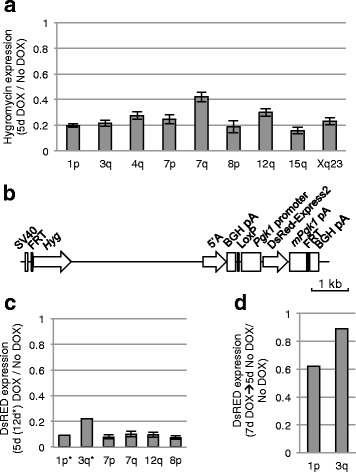
Silencing of flanking reporter genes upon XIST expression from various integration sites. **a** Relative level of *Hyg* expression after 5 days of XIST expression induced by DOX treatment compared with no DOX levels, measured by q-RT-PCR, for each of nine different integration sites as listed. Error bars show the standard deviation of biological triplicates. A one-way ANOVA with Tukey’s Multiple Comparison Test gives the following differences: 1p and 7q**, 3q and 7q**, 7p and 7q*, 7q and 8p**, 7q and 15q***, 7q and Xq** (*P ≤0.05; **P ≤0.01; ***P ≤0.001). **b** Map of transgene containing an inducible construct of the 5’A repeats of *XIST* as well as a DsRed-Express2 reporter gene expressed from a constitutive *Pgk1* promoter. **c** Silencing of DsRed-Express2 relative to no DOX cells, measured by flow cytometry, after 5 or 12 days of DOX induction of 5’A region of *XIST* (from construct shown in part b) that had been integrated into six different integration sites as listed. The error bars represent ±1 s.d. of the silencing levels of individual single-cell clones (*N* = 8–11). **d** DsRed-Express2 expression, measured by flow cytometry, following induction (7 days DOX) and subsequent repression (5 days no DOX) of repeat A of *XIST* at two integration sites (1p and 3q)

We previously reported silencing of a flanking *EGFP* reporter gene at the 3q integration site [20, 40]. At the other FRT sites we did not co-integrate a reporter construct; however, the integration of *XIST* into the FRT site results in expression of an upstream Hygromycin (*Hyg*) gene. Robust silencing of *Hyg* was observed at all integrations after 5 days of XIST expression (Fig. 2a), suggesting that XIST is able to silence a virally-derived promoter (SV40), consistent with our previous demonstration that an EGFP reporter driven by the CMV promoter could be silenced [20]. The 7q integration site showed significantly less silencing than the 1p, 3q, 7p, 8p, 15q, and Xq integration sites (*P* ≤0.01). As only the repeat A region of *XIST* is required for silencing of the flanking reporter genes [40], we generated a construct containing the *XIST* repeat A and a DsRed reporter driven by the mouse *Pgk1* gene promoter, which is normally X-linked and subject to XCI (Fig. 2b). We integrated this construct into six of the integration sites and again observed consistent silencing of greater than 90 % (Fig. 2c), suggesting both viral and mouse-derived promoters could be silenced by XIST in the HT1080 cells. Consistent with previous results with the *EGFP* reporter gene at 3q, silencing of the dsRed reporter was reversible when induction of XIST expression was stopped by removal of DOX (Fig. 2d).

**Fig. 3:**
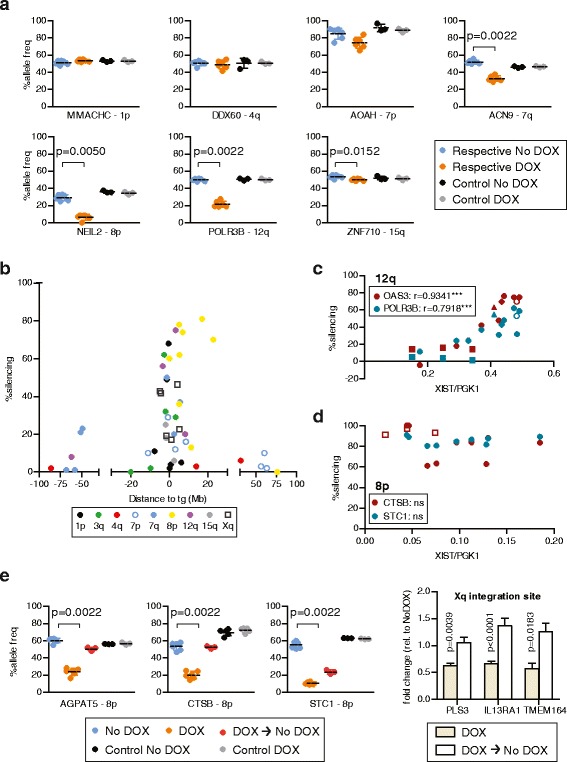
Allelic silencing of flanking endogenous genes upon XIST induction. **a** Allele-discriminating RT-PCR pyrosequencing assay for genes closest to 5 Mb of integration site for each integration site, comparing triplicate cDNAs from untreated cells (No DOX) and following 5-day DOX induction of XIST in duplicate pyrosequencing reactions. cDNA from a different integration was also assessed (additional assays are shown in Additional file 3). *P* values of significantly silenced genes are listed. **b** Summary of the silencing observed for individual genes for each of the nine integration sites (color-coded as shown in the legend); plotted by distance from the integration site on the chromosome (from short to long arm). The allelic change is shown as percent silencing, which was calculated as: (allele frequency No DOX – allele frequency 5d DOX)/allele frequency No DOX × 100) for the pyrosequencing assays. For the Xq integration the silencing was determined by q-RT-PCR since the chromosome is hemizygous. Phase was determined for the 3q integration but for all integrations the allelic change is shown as silencing. Integrations on other chromosomes showed no silencing upon DOX induction. **c** Correlation between extent of silencing and level of XIST RNA. Five different clones (symbols) and cultures show variation in the level of XIST RNA after DOX induction (as measured by qRT-PCR for XIST relative to PGK1), and for 12q this correlates well with the extent of silencing of two genes assayed by allelic pyrosequencing after RT-PCR (OAS3, *P* <0.0001; POLR3B, *P* = 0.0004). A similar analysis (**d**) for the chromosome 8p integration site showed a similar variation in XIST levels, but no correlation with extent of silencing of two loci on 8p. **e** Removal of XIST expression after 5-day DOX induction resulted in substantial reactivation of endogenous genes in the 8p and Xq integration sites

Given the capacity of the XIST RNA to silence in *cis*, and the apparent spread of the RNA along the chromosome based on our RNA FISH data, we questioned whether there would be silencing of endogenous genes at additional sites adjacent to the *XIST* transgenes. The HT1080 cells remain diploid although they carry several structural rearrangements (46,XY,del (1)(p21), i(3)(p10), i(3)(q10), der(4)t(1;4)(p21;p16), der(5)t(5;5)(p15;?), der(11)t(3;11)(q11;q25) see additional details in methods). We generated allele-discriminating pyrosequencing assays to examine silencing of candidate genes flanking the integration sites (Fig. 3). The phase of the polymorphisms relative to the integration was not known, but the allelic expression change upon DOX induction of *XIST* is presented as a *cis*-linked loss of expression as previously demonstrated for the 3q integration for which we were able to assign the allelic loss to the chromosome bearing the inducible *XIST* [40]. Individual pyrosequencing results for the locus closest to 5 Mb from the integration site are shown in Fig. 3a, with the silencing percentages for all genes examined shown in Fig. 3b as a function of distance from the integration site (all assays are shown in Additional file 3). While no significant changes were observed for the control integrations (clones with *XIST* integrated on different chromosomes), all *XIST* integration sites except 4q showed at least one gene with significant allelic silencing. There was much more variability between integration sites for endogenous gene silencing than was seen for the silencing of *Hyg*. The 8p-integrated *XIST* clone displayed the most silencing, with four out of the five genes tested showing 60–80 % silencing. The 4q clone, in contrast, showed no significant silencing for any of the three genes tested. Two different integration sites on chromosome 7 showed quite different results, with only one of seven genes assayed showing over 20 % silencing for the 7p integration site, while five of the seven genes showed over 20 % silencing with *XIST* expressed from the 7q integration site. There was also discontinuous spread of silencing. For example, in the 1p integration site clone, two genes located approximately 200 kb from the *XIST* transgene failed to silence (1 % silencing), whereas the *RHBDL2* gene located approximately 400 kb from the *XIST* transgene silenced by approximately 70 %. In addition to variation between the integration sites in the number of genes that were silenced, there were also significant differences in the extent of gene silencing between genes that showed silencing. More than half of the significant changes demonstrated less than 50 % silencing of one allele, and a significant change as small as 6 % for the *ZNF710* gene on 15q was observed, indicating that XIST can cause a continuum of silencing.

To distinguish differences between clones that are attributable to the integration site rather than clone-to-clone variation, we analyzed clones that were independent integrations of the XIST cDNA into the same genomic location. Intriguingly, for the 12q integration site, we observed a significant correlation between the amount of silencing and the level of *XIST* expression (Fig. 3c). This correlation was observed for both cultures of the same single-cell clone and additional independent clones at the same integration site. Such a correlation was not observed for genes silenced by XIST expression from the 8p integration site using multiple cultures and two independent clones (Fig. 3d). Thus, while variation in the level of XIST expression occurred within a clone, the impact of this variation depended on the integration site, and with similar XIST expression a similar extent of silencing was observed between independent integrations into the same FRT site. A second clone from the Xq integration site showed very similar silencing, while a second clone from the 7q integration site showed lower XIST expression levels and failed to silence (Additional files 1 and 3), suggesting that the silencing ability of 7q integrations, like 12q integrations, might be influenced by XIST expression levels. Since the clones at the 8p integration site silenced across a range of XIST levels, it seemed possible that, unlike silencing of the reporter genes, the maintenance of silencing of endogenous genes might not be XIST-dependent, so we analyzed silencing of endogenous flanking genes after removal of DOX for the 8p and Xq integration sites. All genes examined showed partial to complete reactivation (Fig. 3e) 5 days after induction of XIST had ceased, indicating that the silencing observed requires ongoing XIST expression.

### RNA-seq confirms differences in silencing capacity of XIST at different integrations

The significant differences between the integration sites in their capacity to silence endogenous genes suggested an important impact of the genomic context of the integration; however, it was possible that by chance the genes chosen to be tested were non-random in their ability to silence. Therefore, we chose to examine three clones with *XIST* at different integration sites using RNA-seq to generate a detailed view of any variability in silencing capacity. We chose to examine the 8p and 12q integration sites, which had shown the most silencing, but different sensitivities to XIST levels, as well as the 1p integration site, which had shown limited silencing.

**Fig. 4:**
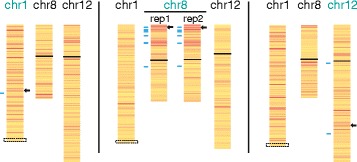
Silencing of endogenous genes upon ectopic XIST expression. Allelic inactivation of chromosomes 1, 8, and 12 is shown as a heat map following DOX induction of XIST in cells containing integrations into the chromosome listed in green. Colors denote the allelic expression change as assessed by RNA-seq (red = downregulation; yellow = no change) for those genes with allelic reads with FPKM ≥5. The integration site is marked as a black arrow; confirmatory pyrosequencing assays are indicated by blue lines, and the centromere is shown as a black line. For chromosome 1, the chromosome is truncated at the site of a translocation as it is unknown whether the integration is on the translocated chromosome (boxed region)

As our candidate genes examined by pyrosequencing had shown significant reductions in gene expression that ranged from as little as 6 % to approximately 80 % silencing of one allele, we did not expect complete silencing of one allele that would reduce expression levels overall by 50 %. We chose a stringent threshold of 30–60 % total reduction to classify genes as silencing, at which level a significantly greater proportion of genes were observed within 30 Mb of the 8p and 12q integration sites compared to the genome (Chi-square test, *P* <0.0001). Consistent with our candidate gene analysis, the increase in proportion of genes in this range was not significant for the 1p integration site. Examination of expression changes as an allelic change is more sensitive to the partial reduction of expression of one allele, and should also still detect changes in allelic expression when the total expression level of the gene is regulated in *trans* although the number of genes that can be examined is reduced by the requirement for an expressed polymorphism. We examined the allelic change in expression on chromosomes 1, 8, and 12 (Fig. 4), and again a significantly higher proportion of genes showing an allelic change of greater than 30 % was seen for genes flanking the 8p integration site. A Chi-square permutation test demonstrated that the number of genes silenced on 8p following XIST induction decreased with increasing distance (*P* = 0.008). The biological duplicate for RNA-seq was highly concordant for the percent allelic gene silencing for 8p (Spearman *r* = 0.5806; *P* <0.0001 for genes with FPKM ≥5). The proportion of genes showing allelic silencing for genes flanking the 1p integration site was significant (*P* = 0.0269); however no significant change was seen for the 12q integration site. We show only the distal end of chromosome 1 as the HT1080 cells carry a translocation of one chromosome 1 to chromosome 4 (approximately 55 Mb distal to the integration site). We validated several of the observed changes by pyrosequencing or q-PCR, and the assays are included in Additional file 3, and highlighted on Fig. 4. Percent allelic silencing was highly concordant between pyrosequencing and RNA-seq (*r* = 0.9341; *P* <0.0001). Of the 17 genes showing at least 50 % allelic silencing in both 8p RNA-seq replicates, two genes (*DLC1* and *STC1*) did not show a decrease in total expression, suggesting that auto-regulation could be another source of discrepancy between total read and allelic read changes. The high rate of validation of the observed changes by pyrosequencing of biological triplicates substantiated that silencing spread to endogenous genes. Intriguingly, on chromosome 8 there appeared to be domains of silencing separated by areas that were more resistant to the action of XIST. In order to explore what features might lead to differential susceptibility to silencing we performed chromatin immunoprecipitation followed by sequencing (ChIP-seq) for induced and uninduced cells with *XIST* integrated at 8p.

### Chromatin features of regions silenced by XIST in somatic cells

**Fig. 5:**
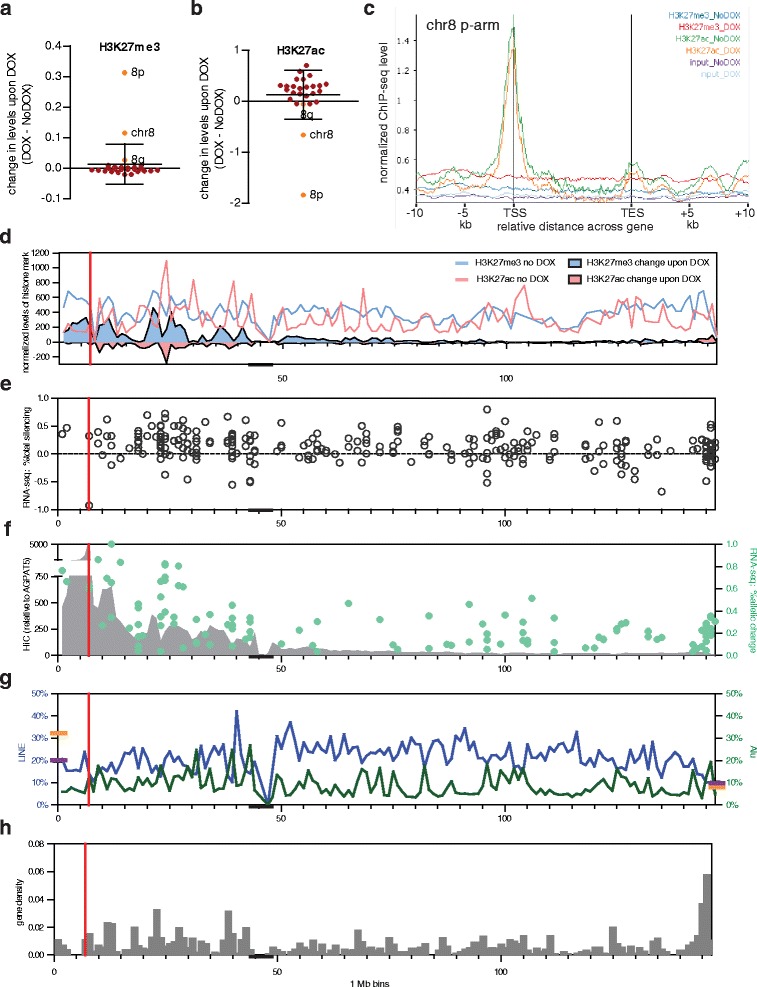
Correlation of genomic neighborhood and XIST-induced silencing on chromosome 8. **a** ChIP-seq changes observed for H3K27me3 on each chromosome, with chromosome 8 also subdivided into 8p (arm with integration) and 8q. H3K27me3 showed significant (*P* = 3.3e^−243^, paired *t*-test) changes for chromosome 8 with the genome showing no significant change. **b** ChIP-seq changes observed for H3K27ac on each chromosome, with chromosome 8 also subdivided into 8p (arm with integration) and 8q. The chromosome 8 decrease (*P* = 2.2e^−40^) as well as the genome-wide increase (*P* = 2.0e^−205^) were both highly significant. **c** Average H3K27ac and H3K27me3 normalized across genes on 8p. Normalized ChIP-seq level are shown across genes and for the 10 kb upstream and downstream of genes before XIST expression (NoDOX) and after XIST expression (DOX) as well as for input, color-coded as outlined. **d** Total H3K27me3 (blue line) and H3K27ac (pink line) in No DOX overlaid with change in H3K27me3 (blue), H3K27ac (pink) along chromosome 8 after 5 days of DOX induction of XIST. **e** Change in total reads for genes with average ≥5 FPKM shown as percent silencing. **f** Density of nuclear contacts with the 8p integration site (AGPAT5) as identified in IMR90 fibroblast cells by HiC [45] in 1 Mb bins (grey shading). Allelic silencing of genes is shown superimposed as green dots. **g** Density of LINE (blue line) and ALU (green line) repetitive elements per 1 Mb bin. Shown on the axes are the average genome (purple) and X-chromosomal (orange) densities of the elements. **h** Gene density in 1 Mb bins along the chromosome

ChIP-seq for both the archetypical facultative heterochromatic mark H3K27me3 and the active mark H3K27ac showed significant, but opposing changes on chromosome 8 upon DOX induction of XIST from the 8p integration site, and these changes were even more dramatic on the short arm where *XIST* is integrated (Fig. 5a, b). Surprisingly, DOX treatment significantly increased H3K27ac across the genome, with most chromosomes showing an increase, suggesting a widespread impact of the antibiotic (Fig. 5b). This unexpected change in chromatin accessibility did not extend to any significant change in H3K27me3 across the genome, and the anticipated enrichments in the imprinted regions of *KCNQ1* and *IGF2R* were observed before and after DOX (Additional file 4). When examined across all genes on chromosome 8p, the loss of acetylation was most notable at the promoter, the site of most pre-existing acetylation, but loss was seen throughout the upstream and gene bodies (Fig. 5c). The genes from 8q showed changes similar to that of other autosomes, with an increase only detected at the promoter (Additional file 5). The gain of H3K27me3 on chromosome 8p was observed across both genic and intergenic regions (Fig. 5c), with no change observed for genes on 8q or autosomes (Additional file 5). In order to correlate the change in H3K27me3 and H3K27ac with the silencing observed, as well as other features of the chromosome, we plotted the initial levels (without DOX) as well as the total change in both marks along the chromosome (Fig. 5d). While the loss of H3K27ac could be anticipated to occur from locations where there was acetylation initially, the recruitment of H3K27me3 upon XIST induction also mirrored the pre-existing levels remarkably well (Spearman *r* = 0.8671; *P* <0.0001 for 8p). In Fig. 5e we show the average silencing determined from total reads, while in Fig. 5f we show allelic gene silencing. We assessed allelic changes in H3K27ac as well, and the limited informative genes showed a significant correlation between allelic silencing and loss of acetylation (correlation for 29 genes with data for both, *r* = 0.5904; *P* = 0.0007). Mouse Xist localizes to sequences that are in contact with the integration site as determined by chromatin conformation capture [43, 44], and therefore we extracted the Hi-C contacts anchored at the 1 Mb domain containing the 8p integration site from the published Hi-C data [45] (Fig. 5f). There are more contacts, as well as stronger silencing, closer to the integration site, confounding the ability to examine correlations. Interestingly, the Hi-C contacts correlated with the pre-existing (No DOX) H3K27me3 levels along the chromosome 8 short arm (Spearman *r* = 0.4996; *P* = 0.0006), although we did not identify an association of allelic silencing with domains designated as closed nor open [46] (Fisher’s exact test). Allelic silencing correlated with both the gain of H3K27me3 (Spearman *r* = 0.4599; *P* = 0.0003) and loss of acetylation (Spearman *r* = −0.4557, *P* = 0.0004). Given the proposed role for repetitive elements in the XCI process [47–49], and the tendency for mouse Xist to first localize to gene-rich regions during early expression in embryonic stem cells [43, 44] we show the LINE and ALU distribution (Fig. 5f) and the gene density (Fig. 5g) along chromosome 8; however, neither feature showed a significant correlation with silencing, although as would be expected, gene density is a major contributor to total levels of H3K27 acetylation. To further explore the relationship of the ability to modify chromatin and the silencing ability of the different chromosomal integration sites we performed immunofluorescence in conjunction with FISH for XIST.

### Recruitment of heterochromatic modifications to the site of XIST localization

**Fig. 6:**
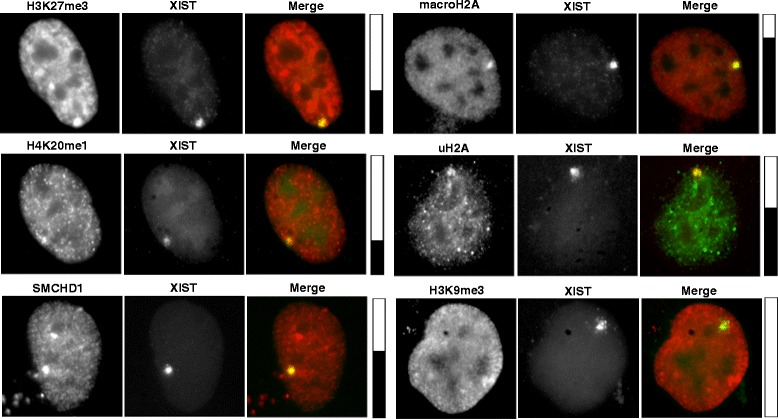
Immunofluorescence shows features enriched at site of XIST RNA. The XIST RNA is identified by RNA FISH (green, except for H2AK119u1 (uH2A) photos, shown in greyscale) after 5 days DOX induction for the Xq integration clone. Co-IF with the antibodies listed was performed (shown in grayscale), with the merged image in color. Bars to the right show the proportion of XIST-positive HT1080 cells (integration into Xq) displaying enrichment of the indicated chromatin modification/protein

In addition to H3K27me3, the Xi is known to be enriched for additional histone marks and proteins associated with repressed chromatin; however, the hierarchy of recruitment of these heterochromatic features by XIST has not been clarified. Therefore, we examined the co-localization of these features with XIST expressed from various integration sites (Table 2). The extent of co-localization can vary with the cell cycle, and therefore we categorize the co-localization as positive (consistently observed in greater than 25 % of XIST-positive cells), negative (consistently observed in less than 10 % of cells) or +/− (in between 10 % and 25 % co-localization, or variable between replicates). No recruitment of H3K9me3 was observed at the four sites examined, while H2AK119u1 was observed at all four examined integration sites. The integration onto Xq showed recruitment of all marks (with the exception of H3K9me3) and is shown in Fig. 6. Recruitment was still less than was seen for a female cell line – in IMR90 we routinely detect over 80 % co-localization. H3K27me3 was not recruited to the 1p or 3q integration sites, while SMCHD1 was not enriched at 1p, 3q, and also 4q integration site clones. The integration clone at 3q did however recruit macroH2A, which was not seen to be enriched at the 1p or 7q integration clones. Overall the integration at 1p showed the least enrichment of marks with only variable recruitment of H4K20me1 in addition to H2AK119u1. To further examine dependence on integration site, independent clones integrated into 7q, Xq, and 8p were compared by IF-FISH for H3K27me3 and/or H4K20me1. Of the six side-by-side comparisons of independent clones at the same integration site, enrichment of marks were very comparable showing an average of 6 % difference in enrichment and all falling within the same category (except for 8p with H4K20me1 which spanned the +/− and +).

**Table 2:** Co-localization of histone modifications with induced XIST RNA signal

Clone	H3K27me3	H4K20me1	macroH2A	SMCHD1	H2AK119u1	H3K9me3
1p	−	+/−	−^a^	−	+	−
3q	−		+^a^	−^a^		
4q	+/−	−	+/−	−^a^		
7p	+/−	+/−	+	+/−^a^		
7q	+/−^b^	−^b^	−^a^	+/−		
8p	+^b^	+/−^b^	+ ^a^	+^a^	+	−
12q	+/−^b^	−^a^	+	+/−	+	−
15q	+/−	+/−	+/−	+/−		
Xq	+	+^b^	+^a^	+	+	−

^a^In situ hybridization performed at least in duplicate

^b^In situ hybridization performed on two independent clones

Co-localization of the histone modification with the XIST signal was counted in at least 30 cells. No co-localization (−) was <10 % co-localization; +/− was between 10 % and 25 % co-localization and + is assigned to integration sites with over 25 % co-localization

The only mark examined that was not detected at any site was the H3K9me3 mark, which is often associated with constitutive heterochromatin. H2AK119u1 was seen at all of the four integrations examined, suggesting it is less dependent on local chromatin structure than the other marks which were heavily dependent upon the integration site and surprisingly independent of each other. The X chromosome showed the best ability to recruit all features examined, with the next best integration site also being the one that showed the greatest gene silencing (8p).

## Discussion

We have examined the impact of genomic location of XIST on its ability to alter nuclear ultrastructure, chromatin state, and gene expression in a somatic cell line. We observe considerable heterogeneity in the extent of silencing and the recruitment of chromatin marks, depending upon the integration site, providing us with an opportunity to dissect the interactions between features in a system in which complete recruitment and silencing does not occur. The ability of XIST to localize to all integration sites supports that there is limited sequence specificity for the RNA with the chromosome, in agreement with the spread of inactivation reported in X/autosome translocations, and various mouse *Xist* transgenes (reviewed in [36, 50]). The lack of sequence specificity is also in line with the recently described proximity-transfer model which suggests that Xist first associates with sequences in physical proximity to the *Xist* locus and then transfers to gene-rich regions which are topologically associated with the integration site [43].

Spread of silencing to autosomes in these HT1080 cells is not seen to the extent that is observed for X/autosome translocations [48, 49] or an autosomal *XIST* transgene in iPS cells [39], suggesting that bypassing differentiation reduces the ability to inactivate a chromosome. While early mouse studies suggested the presence of a limited developmental window during which silencing could be induced [12] we see XIST-dependent silencing of endogenous genes in these HT1080 fibrosarcoma cells up to almost 50 Mb from the integration site, although endogenous gene silencing is most prominent closer to the integration site. It is possible that the cancerous origin of these cells has reactivated critical developmental gatekeepers, as specific mouse cancers have been shown to allow Xist-induced silencing [17, 25]. The ability of XIST to induce gene silencing in somatic cells has important implications for cancer cells where rearrangements or reactivation of XIST may bring previously active genes under the influence of XIST.

**Fig. 7:**
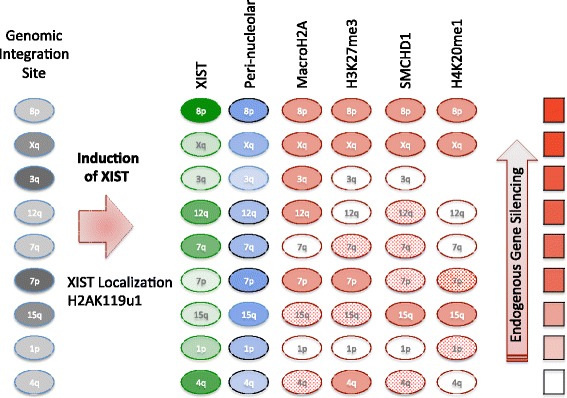
Schematic of features examined at the site of XIST RNA induction. Nine different integration sites of XIST were examined, and these were in both G-light (pale gray) and G-dark genomic locations. Upon DOX induction XIST was expressed (intensity of green oval reflects average amount of XIST expression) and increased perinucleolar localization was observed (blue oval intensity reflects increase, with significant changes encircled in black). H2AK119u1 was enriched at all four integration sites examined. The enrichment of chromatin marks or proteins that were variably recruited (see Table 2) is shown as solid (enrichment >25 %), dotted (enrichment between 10 % and 25 %) or unfilled (enrichment ≤10 %). The integration sites are ordered by ranking of gene silencing observed by pyrosequencing (fill of red rectangle reflecting proportion of silenced genes, see Additional file 3)

We observe a Cot-1 hole and increased perinucleolar association at all integration sites, consistent with previous reports that XIST/Xist expression from autosomes increases perinucleolar association [10, 21]. In our assessment of multiple XIST integrations, we observed heterogeneity in the extent of perinucleolar association, and the correlation with silencing was limited. The three integration sites for which the increase in perinucleolar association was not significant (15q, 3q, and Xq) were all G-dark integration sites; however, the 7p integration was also in a G-dark band yet demonstrated a significant increase in perinucleolar localization, suggesting an incomplete association between perinucleolar association and G-dark integrations with lower XIST levels (Fig. 7).

The other feature that we observed at all integrations examined was H2AK119u1, which is established by the PRC1 complex. We observed H2AK119u1 in the absence of SMCHD1, macroH2A, and H3K27me3 in the 1p integration cells, suggesting that XIST may be able to directly recruit the PRC1 complex, in agreement with the PRC2-independent PRC1 recruitment previously suggested in mice [27]. As the 1p integration site demonstrated limited silencing in the presence of H2AK119u1 recruitment, we conclude that H2AK119u1 is insufficient for the spread of gene silencing. No integration site examined showed recruitment of H3K9me3, although the antibody clearly hybridized to the native Xi in female cells. Therefore it appears that facultative heterochromatic marks can be recruited by XIST in these cells, but the establishment of a ‘locked-in’ silent state required additional layers of developmentally-regulated chromatin condensation. Without such locks on silencing, the continued silencing of both reporter genes and endogenous genes was XIST-dependent, undergoing reactivation upon removal of XIST induction.

Interestingly, no single factor was seen to associate with more robust silencing (Fig. 7), demonstrating considerable redundancy in XIST-inducible silencing pathways. Consistent with previous studies in mice, silencing of endogenous genes in these human cells was observed in the absence of recruitment of macroH2A (7p) [30], SMCHD1 (3q) [51], or H3K27me3 (12q) [27]. MacroH2A has been reported to be recruited by induction of mouse *Xist* expression [13], and is also lost upon deletion of *Xist* [14]; yet intriguingly we observe macroH2A recruitment to be site-dependent, rather than solely *XIST*-dependent. We observed that macroH2A is not required for XIST-induced silencing, which is consistent with the ability of mice that are knocked-out for both macroH2A1 and macroH2A2 to continue to undergo XCI [30]. The recruitment of SMCHD1 and macroH2A seems to be independent of each other as, for example, the 3q integration site recruits macroH2A but not SMCHD1, while the 7q integration shows some SMCHD1 recruitment, but no macroH2A recruitment. There was a positive correlation between the amount of SMCHD1 recruitment and the final perinucleolar localization (Spearman *r* = 0.8; *P* = 0.01), with the proportion of cells positive for SMCHD1 always equal or lower to the percent seen to be perinucleolar. Thus perinucleolar localization may be required but not sufficient to recruit SMCHD1. The strong ability of the X integration to recruit all features confounded the ability to detect correlations; however, it appears that the majority of the features are independently recruited to the chromosome that expresses XIST. For several of the integration sites two or more independent clones with similar XIST levels behaved similarly supporting that the variation we observe is due to the integration site. In addition, the 4q and 12q clones were derived from a different subclone of HT1080 from the other autosomal integration sites; however, they did not appear to be more similar to each other, arguing against the variation arising during subclone generation.

The differential recruitment of chromatin marks depending on integration site suggests that multiple silencing pathways not only work in parallel to promote silencing, but that their recruitment is favored by different underlying DNA sequences, consistent with previous studies using genes that escape from inactivation to identify 12 features of the DNA sequence of the X chromosome that may be involved in the spread or maintenance of XCI [52].

## Conclusions

Overall, we saw variability in the recruitment of chromatin marks between the integration sites, highlighting the importance of the integration site in modulating XIST function. Localization of XIST with a concomitant depletion of Cot-1 RNA, recruitment of H2AK119u1, and a shift to perinucleolar location was seen at all integrations, and thus reflect features established by XIST independent of the local chromatin environment. In contrast, recruitment of SMCHD1, macroH2A, H3K27me3, and H4K20me1 appeared to be strongly influenced by the site of XIST expression. As some silencing was observed at all integrations, this study demonstrates that silencing in human somatic cells can occur in the absence of macroH2A, SMCHD1, and H3K27me3/H4K20me1 recruitment, underscoring the independent but cooperative nature of the X-chromosome inactivation process. The X chromosome demonstrated the most consistent ability to recruit the heterochromatic marks of XCI, consistent with an evolutionary accumulation of DNA features enabling the recruitment of heterochromatic marks to the X chromosome.

## Methods

### Generation and culture of cell lines and identification of the transgene integration site

HT1080 HH1 cells were transfected with pcDNA6/TR, and two subclones (HT1080HH1-2-3 or 2–12) expressing high RNA levels of the Tet-repressor were subsequently transfected with pFRT/*lac*Zeo (Life Technologies) at low concentrations. The full-length inducible *XIST* cDNA construct [20] was co-transfected with the pOG44 plasmid expressing Flp recombinase for site-specific recombination into the FRT site followed by Hygromycin selection and confirmation of loss of Zeomycin resistance. Cells were grown at 37 °C with 5 % CO_2_ in DMEM supplemented with penicillin/streptomycin, non-essential amino acids, and 10 % V/V fetal bovine serum. XIST expression was induced with the addition of 1 μg/mL doxycycline to the culture medium.

Southern blotting identified eight FRT integrations that appeared single copy. Inverse PCR utilizing primers complementary to a sequence within the integrated pFRT plasmid was used to identify the precise integration site of the transgenes in the HT1080 cell lines. The ends of linearized plasmids are subject to exonuclease activity, thus the actual integrated transgene often lacks several hundred of base pairs on each end which was first identified by a series of PCR assays prior to restriction endonuclease digestion with a frequently-cutting restriction endonucleases identified to cut in the remaining plasmid, followed by ligation with T4 DNA ligase (Invitrogen) to create circular DNA molecules. The captured genomic DNA was amplified by nested PCR with primers facing outward from the plasmid fragment for sequencing and the genomic location was identified using the BLAT algorithm [53]. No X-linked integration was identified, so the F55 HT1080 clone from Yan and Boyd was used [41].

While cancer derived, the HT1080 cells remain diploid with four structural rearrangements detected by spectral karyotyping (46,XY,del (1)(p21), i(3)(p10), i(3)(q10), der(4)t(1;4)(p21;p16), der(5)t(5;5)(p15;?), der(11)t(3;11)(q11;q25). Using our allelic pyrosequencing assays we observed instability of chromosome 3 in two of nine clones (see also [40]) and homozygosity for assays on 4q in one clone, while assays on chromosomes 1, 7, 8, and 15 remained diploid in the clones tested, suggesting that the unbalanced rearranged chromosomes were the least stable in these cells. We analyzed two independent clones at the Xq, 7q, and 8p integration site and five independent clones of the 8p integration site. These clones were individual single-cell clones following Flp-mediated recombination into the FRT site. Each clone was selected for *Hyg*-resistance and assessed for loss of Zeomycin sensitivity.

As fluorescent reporters allow for efficient screening, we created a plasmid that carries both the inducible repeat A and a DsRed-Express2, driven by the mouse *Pgk1* promoter (Fig. 2b). To test whether the ability of repeat A to silence the reporter depends on the genomic integration site, we inserted the repeat A – DsRed-Express2 transgene into six of the HT1080 cell lines with a known chromosomal location of the FRT integration site for assessment of silencing by flow cytometry as previously performed [40].

### RNA FISH and immunofluorescence

Cells were grown on glass coverslips. Upon removal from cell culture the coverslips were first rinsed in ice-cold CSK buffer (0.3 M sucrose, 100 μM NaCl, 10 μM PIPES, 3 μM MgCl_2_), then permeabilized with 0.5 % Triton-X 100 in CSK for 8 min on ice and then fixed in 4 % paraformaldehyde for 8 min at room temperature. Coverslips were stored at 4 °C in 70 % ethanol. Just prior to RNA FISH, the coverslips were immersed in 100 % ethanol for 5 min and left to air dry. FISH was performed with two probes: an XIST probe and a Cot-1 (Invitrogen) probe. Both probes had been directly fluorescently labeled using the Nick Translation Reagent Kit (Abbott Molecular, Inc.) with Spectrum red-UTP (Vysis) for Cot-1 DNA probes and Spectrum green-UTP (Vysis) for XIST probes. Approximately 150 ng of each probe was mixed together along with 20 μg salmon testes DNA then air dried in a speed vacuum, resuspended in 10 μL deionized formamide, denatured at 80 °C for 10 min, and then mixed with 10 μL hybridization buffer (20 mg/mL BSA, 4XSSC, 20 %). This was pipetted onto a small square of Parafilm and the coverslip was placed on top of the probe mixture. Another piece of Parafilm was then placed on top and the edges were sealed to prevent the drying out of the coverslip. Hybridization took place overnight in a humidified chamber at 37 °C. The next day the coverslips were rinsed as follows: 20 min in 50 % formamide/50 % 4XSSC at 37 °C, 20 min in 2X SSC at 37 °C, and 20 min in 1X SSC at room temperature. Coverslips were then stained with DAPI and mounted onto microscope slides with Vectashield (Vector Laboratories). Cells were observed on a Leica inverted microscope (DMI 6000B) at 100X magnification and images were obtained using a Retiga 4000R (Q-Imaging) camera with Openlab software (PerkinElmer). Images were processed using Adobe Photoshop CS4 to reduce background and correct for variation in FISH efficiency between different images. A one-way ANOVA test in GraphPad was used to determine significantly different signal sizes. Line scans were generated using Image J software (NIH) by drawing a line through the area of interest and plotting the RGB intensities across the line.

To determine the nuclear location, XIST signals were scored visually in Photoshop as being either ‘perinuclear only’ (for example, adjacent to and in contact with the nuclear periphery), ‘perinucleolar only’ (that is, adjacent to and in contact with a Cot-1 negative nucleolus), ‘both’ (that is, adjacent to and in contact with both the nuclear periphery and a Cot-1 negative nucleolus), or ‘neither’. XIST signals scored as ‘both’ are included in the ‘perinuclear’/‘perinucleolar’ percentages in Table 1. For the ‘5d DOX’ counts, results for each integration site are the average of three independent experiments performed on different coverslips, by at least two independent observers, with a minimum of 50 cells counted each time. The ‘No DOX’ counts were done once with a minimum of 60 cells counted per integration site.

For combined RNA FISH and immunofluorescence, coverslips, which had been stored at 4 °C in 70 % ethanol, were first rinsed in PBS then placed onto a small amount of PBT (PBS with 1 % BSA and 0.1 % Tween 20) containing 0.4 U/μL Ribolock RNase inhibitor. Coverslips were sealed between two layers of Parafilm and left in the blocking buffer at room temperature for 20 min, then transferred from blocking buffer to PBT containing 1:100 primary antibody and 0.4 U/μL Ribolock, sealed between two layers of Parafilm and left at room temperature for 4–6 h. Coverslips were then washed three times, for 5 min each time, at room temperature in PBS containing 0.1 % Tween 20, then put onto a small amount of PBT containing 1:250 fluorescently labeled secondary antibody and 0.4 U/μL Ribolock, sealed between two layers of Parafilm and left at room temperature in the dark for 45 min. Coverslips were then washed three times, for 5 min each time, at room temperature in the dark in PBS containing 0.1 % Tween 20. Coverslips were then fixed in 4 % PFA in PBS for 10 min at room temperature in the dark, and washed for 5 min in PBS before continuing on to RNA FISH, making sure that the coverslips remained in the dark throughout the RNA FISH procedure. Antibodies used in immunofluorescence include: anti-H3K27me3 (07–449 from Millipore); anti-macroH2A (07–219 from Millipore); anti-SMCHD1 (ab31865 from Abcam); anti-H2AK119u1 (05–678 from Millipore); anti-H3K9me3 (07–442 from Millipore); anti-H4K20me1 (07–440 from Millipore).

### RNA isolation, reverse transcription Q-PCR and allelic discrimination by pyrosequencing

RNA was isolated from cell pellets stored at −70 °C using TRIZOL (Invitrogen) according to the manufacturer’s instructions and then treated with DNase1. cDNA was generated in the range of 0.5–2.5 μg RNA using M-MLV reverse transcriptase for qPCR on a StepOnePlusTM Real-Time PCR System (Applied Biosystems, Darmstadt, Germany), using Maxima Hot Start Taq (Thermo Scientific) and EvaGreen dye (Biotium). The following conditions were used: 95° for 5 min, followed by 40 cycles of (95° for 15 s, 60° for 30 s, 72° for 1 min), and a melt curve stage of (95° for 15 s, 60° for 1 min, increase of 0.3° until 95°). The expression levels of genes of interest were normalized to the expression of *ACTB* or *PGK1*. Primer sequences are found in Additional file 6.

Pyrosequencing of cDNA before and after DOX induction of DNA, and of clones containing integrations on alternate chromosomes were examined. Each 25 μL PCR was performed with 1x PCR Buffer (Invitrogen), 0.2 mM dNTPs, 0.625 U *Taq* DNA polymerase (Invitrogen), 0.5 μM forward and reverse primers, and 50–100 ng of cDNA for (94C for 30 s, 58.3C for 30 s, 72C for 1 min) × 35 cycles, and 72 °C for 10 min for final extension. One of the forward and reverse primers was biotinylated for template isolation during pyrosequencing preparation. Universal M13 primer was also used for some assays (see Additional file 6), where the primer to be biotinylated instead contained the M13 sequence at the 5′end (5′CGC CAG GGT TTT CCC AGT CAC GAC3′). Nested PCR was run for the assays that utilized the universal primers: the first round of PCR was performed with the same cycling conditions but only for 15x cycles, with the M13-tagged primer (without the biotin) and its paired forward or reverse primer; the second round of PCR was run with 1 μL of PCR product from the first round of PCR as the template, as well as the biotinylated M13 primer and the non-M13-tagged primer that was used in the first round, under the same cycling condition but for 20 cycles. Pyrosequencing was performed on the PyroMark MD machine (Qiagen). Template preparation for pyrosequencing was done according to manufacturer’s protocol. For each assay, 10–15 μL of PCR products was used as template and CDT tips were used to dispense the dNTPs.

### Sequencing analysis

For both ChIP-seq and RNA-seq, library preparation and sequencing was done according to Illumina protocols, and reads were aligned to hg18 reference genome using Tophat. RNA-seq was performed as previously described [54], first on two 8p clones following 5 days DOX and the 8p clone with No DOX producing 50 bp paired-end reads, with a Pearson correlation between replicate DOX FPKM values of *r* = 0.9934 (*P* <0.0001). A second RNA-seq of the 12q and 1p integration sites with No DOX and following 5 days DOX produced 76 bp paired-end reads. Total RNA expression was quantified in FPKM using Cufflinks. ChIP of H3K27me3 (Active Motif antibody 61017) and H3K27ac (Active Motif antibody 39133) was performed on the 8p clone with and without DOX treatment, as previously done [55] using 20 μg of chromatin and 3 μg of antibody per IP. ChIP-seq produced 36 bp single-end reads, and the ChIP-seq data were analyzed using SeqMonk to identify enriched H3K27ac peaks (MACS, *P* value = 1 × 10^−3^) and to quantify the level of H3K27me3 in 2 kb and 1 Mb non-overlapping windows.

Allelic information for both RNA-seq and ChIP-seq was obtained with a custom workflow in Galaxy [56–58]. Briefly, mapped reads were run through Samtools mpileup to obtain SNPs relative to the reference genome in the control and experimental (integration of interest) samples, and allelic ratio of reads was calculated at each variant. In addition, only the variants with a biallelic ratio of 0.3–0.7 in the No DOX (control) sample were further examined. In order to phase the variants, the allele with lower reads for each variant site in the DOX (experimental) sample was considered to be silenced in *cis* with the *XIST* transgene. Variant sites were next combined based on regions of interest, either gene location or genomic regions for RNA-seq and ChIP-seq, respectively. Variant sites that had an allelic ratio 0.15 greater or less than the overall gene allelic ratio for the sample were removed and a new overall gene ratio was calculated. For ChIP-seq, variants with reads >2-fold different from the average number of reads in the region of interest were also excluded. Percent allelic silencing for a given gene or region of interest was defined as (control allelic ratio – experimental allelic ratio)/control allelic ratio. The calculated ratio was the frequency of the allele with lower reads in the experimental sample for each gene or region of interest. We also required that there were at least 4 reads and 5 reads with allelic information per gene (RNA-seq) and region of interest (ChIP-seq), respectively, in both the control and experimental samples to be considered for analysis.

### Hi-C analysis

We obtained normalized Hi-C data for human female fibroblast line IMR90 [45] and the Hi-C analysis was done as previous [43]. We calculated the 1 Mb Hi-C counts by summing the counts in all 40 kb bins within each 1 Mb bins across the chromosome, with the anchor being the 1 Mb bin containing the *XIST* transgene (*AGPAT5* for 8p clone).

### Data availability

The sequence data described are available in GSE68109. Additional microscopy images are available in Figshare (http://dx.doi.org/10.6084/m9.figshare.1529822).

## Additional files

Additional file 1:
**Genomic location of FRT integration sites in the HT1080 cell lines.** (DOCX 129 kb) Additional file 2:
**Location of XIST RNA signal for each integration site with and without DOX treatment.** The numbers shown are based on three independent experiments for the DOX results and one experiment for the No DOX results, with ≥50 cells counted for each integration site in each experiment. (PDF 57 kb) Additional file 3:
**Candidate gene silencing assays for each integration site.** One assay for each integration site was included in Fig. 2. **a** Allelic pyrosequencing of three cDNAs from No DOX and DOX were compared in duplicate pyrosequencing reactions. As a control cDNA from a different integration was also assessed. **b** For the X-chromosome integration site q-RT-PCR was used to determine silencing as the cells are hemizygous. **c** Q-RT-PCR assays were also performed to validate some autosomal silencing and compare with pyrosequencing and RNA-seq. (PDF 933 kb) Additional file 4:
**Imprinted regions showed broad enrichment of H3K27me3 and punctate peaks of H3K27ac.** Probes with values in the extreme 5 % of inputs were removed. (PDF 175 kb) Additional file 5:
**Average H3K27ac and H3K27me3 for genes on 8q and chromosome 1.** The normalized ChIP-seq level are shown across an aggregate of genes and for the 10 kb upstream and downstream before (NoDOX) and after XIST expression (DOX). (PDF 246 kb) Additional file 6:
**Table of primers.** (XLSX 16 kb)
