# Supplementary material for: Impact of flanking chromosomal sequences on localization and silencing by the human non-coding RNA XIST
Source: Genome Biol. 2015 Oct 2;16:208. doi: 10.1186/s13059-015-0774-2 (PMC4591629; doi:10.1186/s13059-015-0774-2)
Supplement: Additional file 1: — Genomic location of FRT integration sites in the HT1080 cell lines. (DOCX 129 kb) [file 13059_2015_774_MOESM1_ESM.docx]

Additional Data File 1: Genomic location of FRT integration sites in the HT1080 cell lines.

| **Integration site** | **Cell designation^a^** | **G-band** | **Distance to**  **nearest gene** | **XIST Expression**  **(FPKM)**  **No Dox 🡪 DOX** |
| --- | --- | --- | --- | --- |
| 1p34.3 | 2-3-1.0d  2-3-1.0d-1C | light | Intronic  (MACF1) sense | 4.2 🡪 22.0 |
| 3q28 | 2-3-0.5+3#4  2-3-0.5+3#4SCC3A.1 | dark | 10 kb  (CLDN1) | 1 🡪 10 ** |
| 4q32 | 2-12-0.5#8  2-12-0.5#8SCC5.1 | light | 55 kb  (DCHS2) | 1 🡪 60 ** |
| 7p14.3 | 2-3-1.0#5  2-3-1.0#5-2Bi | dark | 20 kb  (BBS9) | 0.7 🡪 9.8 |
| 7q21.2 | 2-3-0.5+3#1  2-3-0.5+3#1clone1  2-3-0.5+3#1clone7 | light | 215 kb  (MTERF) | 1.7 🡪 55  1 🡪 35 ** (alt. clone) |
| 8p23 | 2-3-0.5a  2-3-0.5aSCC1C.1  2-3-0.5aSCC1A.1 | light | Intronic  (AGPAT5) sense | 3.4 🡪 94.4  1 🡪 50 ** (alt. clone, see Fig. 3d for range) |
| 12q24 | 2-12-1.0#14  2-12-1.0#14clone2A.2  also clones 1A.1; 3A.2; 3A.3; 5A.1 | light | Intronic  (FAM222A) antisense | 3.8 🡪 67.6  1 🡪 35-70 ** (alt. clones, see Fig. 3c for range) |
| 15q | 2-3-1.0#12  2-3-1.0#12clone3A | dark | 89 kb  (LINC00052) | 1 🡪 10 ** |
| Xq23 | F 55-6B1  F 55-6B1-N6  F 55-6B1-N1 | dark | 150 kb  (HTR2C) | 3.3 🡪 17.7 **  1 🡪 15 ** |

**^a^**The clones beginning with 2-3 and 2-12 were derived from independent integrations of the pcDNA6/TR vector for constitutive expression of the Tet repressor. The F55 cell line was independently derived as described [[41](#_ENREF_39)]. The first row is the designation for the line with the FRT site, the second row the clone with the full XIST cDNA VI.34 integrated into the FRT site. Additional independent clones (alt. clone) examined are listed in blue. **Q-RT-PCR estimate of XIST expression relative to FPKM of cells derived from RNA-seq.
