# Supplementary material for: Impact of flanking chromosomal sequences on localization and silencing by the human non-coding RNA XIST
Source: Genome Biol. 2015 Oct 2;16:208. doi: 10.1186/s13059-015-0774-2 (PMC4591629; doi:10.1186/s13059-015-0774-2)
Supplement: Additional file 2: — Location of XIST RNA signal for each integration site with and without DOX treatment. The numbers shown are based on three independent experiments for the DOX results and one experiment for the No DOX results, with ≥50 cells counted for each integration site in each experiment. (PDF 57 kb) [file 13059_2015_774_MOESM2_ESM.pdf]

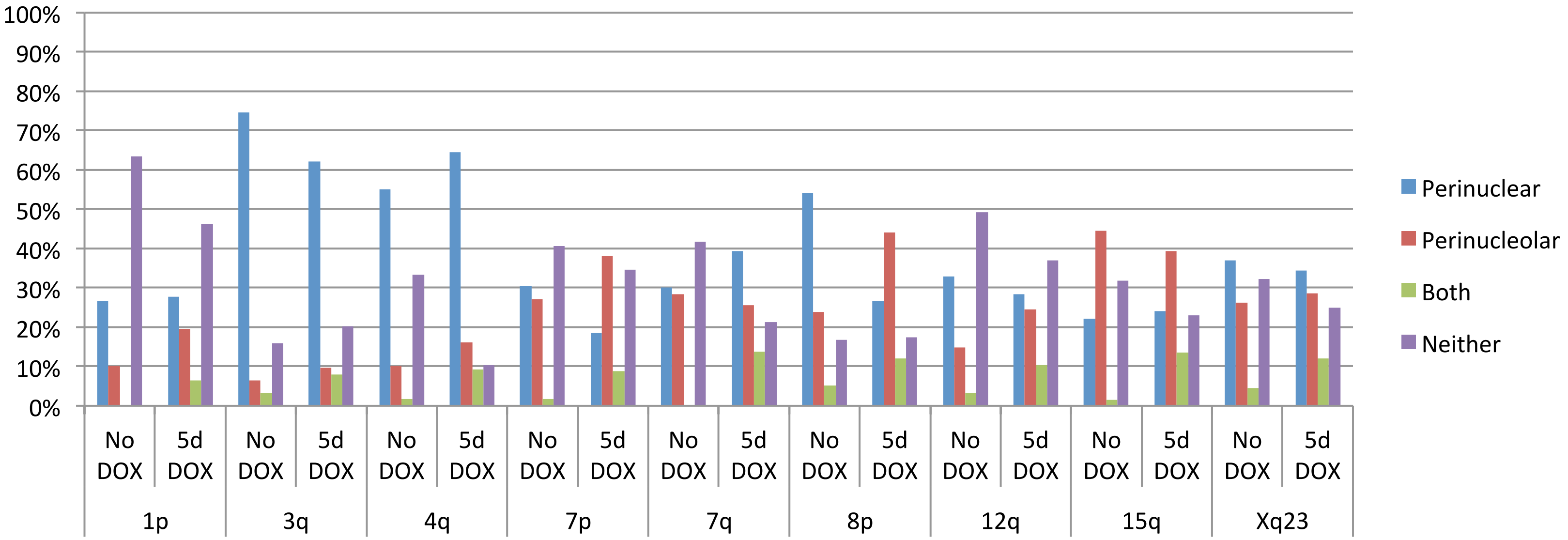


Additional Data File 2: **Location of XIST RNA signal for each integration site with and without DOX treatment.**

The numbers shown are based on three independent experiments for the DOX results and one experiment for the No DOX results, with ≥50 cells counted for each integration site in each experiment.
