# Supplementary material for: Impact of flanking chromosomal sequences on localization and silencing by the human non-coding RNA XIST
Source: Genome Biol. 2015 Oct 2;16:208. doi: 10.1186/s13059-015-0774-2 (PMC4591629; doi:10.1186/s13059-015-0774-2)
Supplement: Additional file 3: — Candidate gene silencing assays for each integration site. One assay for each integration site was included in Fig. 2. a Allelic pyrosequencing of three cDNAs from No DOX and DOX were compared in duplicate pyrosequencing reactions. As a control cDNA from a different integration was also assessed. b For the X-chromosome integration site q-RT-PCR was used to determine silencing as the cells are hemizygous. c Q-RT-PCR assays were also performed to validate some autosomal silencing and compare with pyrosequencing and RNA-seq. (PDF 933 kb) [file 13059_2015_774_MOESM3_ESM.pdf]

Additional Data File 3. Candidate gene silencing assays for each integration site.

a

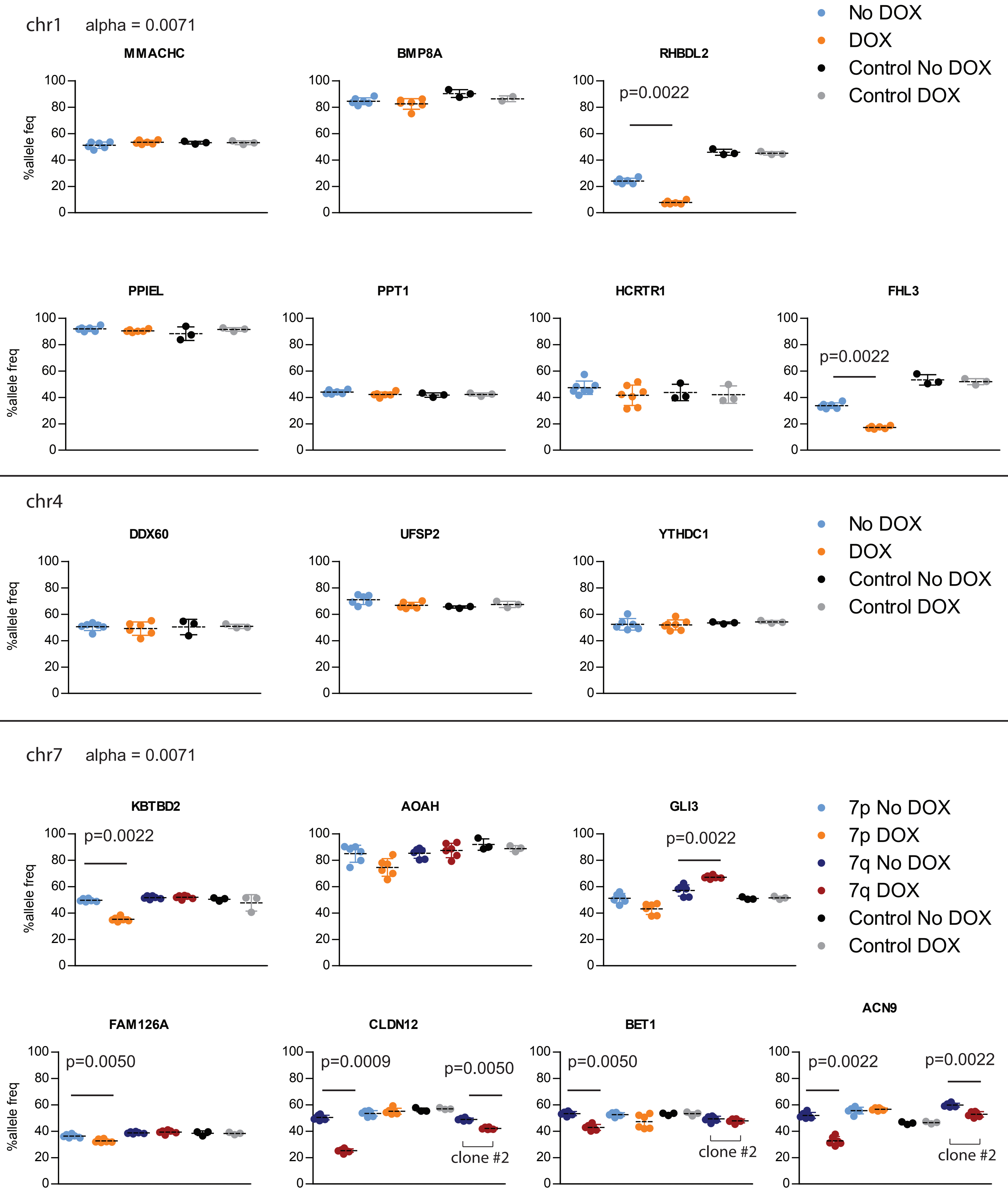

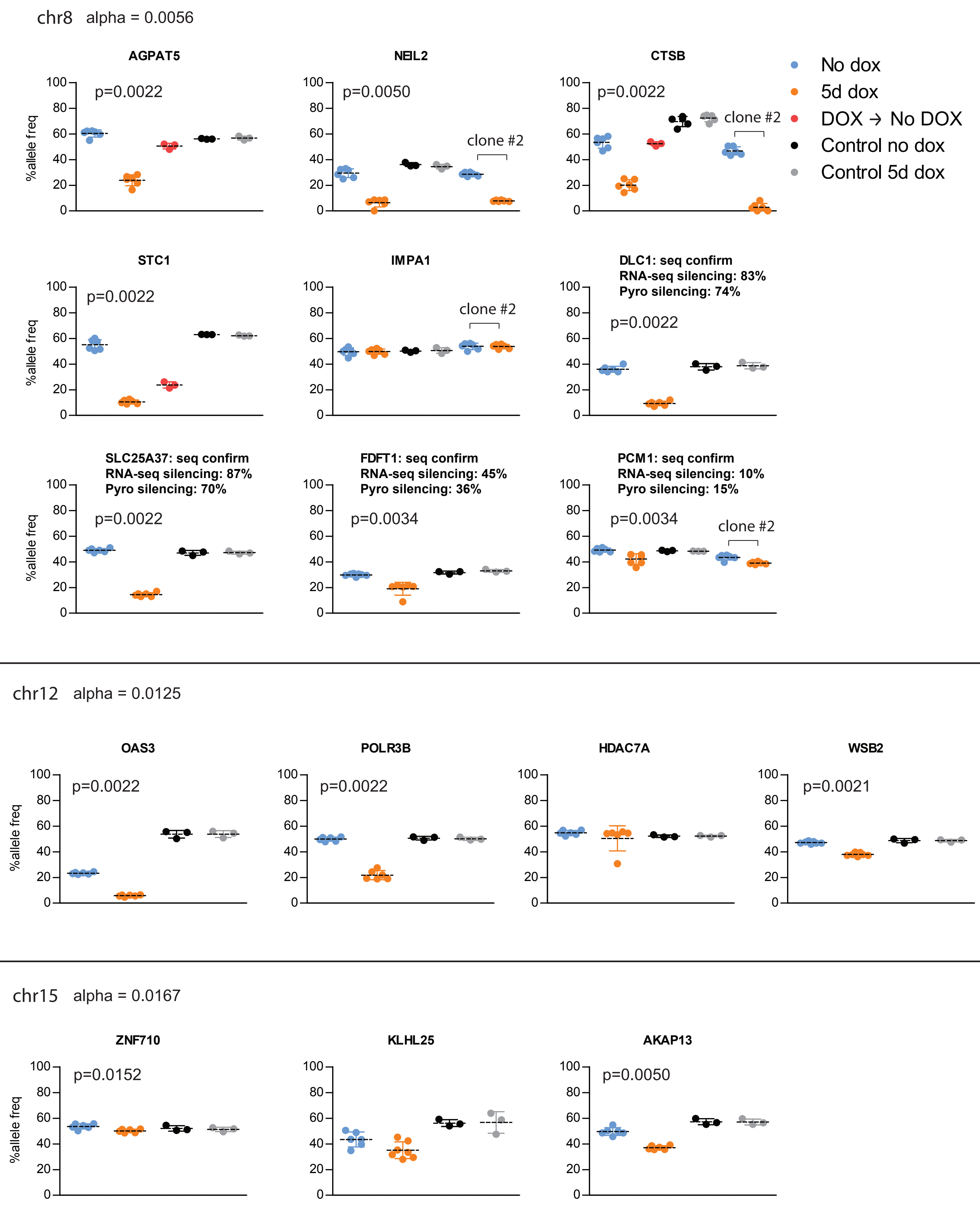

chr8 alpha = 0.0056
AGPAT5
p=0.0022
NEIL2
p=0.0050
clone #2
CTSB
p=0.0022
clone #2
No dox
5d dox
DOX → No DOX
Control no dox
Control 5d dox
STC1
p=0.0022
IMPA1
clone #2
DLC1: seq confirm
RNA-seq silencing: 83%
Pyro silencing: 74%
p=0.0022
SLC25A37: seq confirm
RNA-seq silencing: 87%
Pyro silencing: 70%
p=0.0022
FDFT1: seq confirm
RNA-seq silencing: 45%
Pyro silencing: 36%
p=0.0034
PCM1: seq confirm
RNA-seq silencing: 10%
Pyro silencing: 15%
p=0.0034
clone #2
%allele freq
chr12 alpha = 0.0125
OAS3
p=0.0022
POLR3B
p=0.0022
HDAC7A
WSB2
p=0.0021
chr15 alpha = 0.0167
ZNF710
p=0.0152
KLHL25
AKAP13
p=0.0050

b

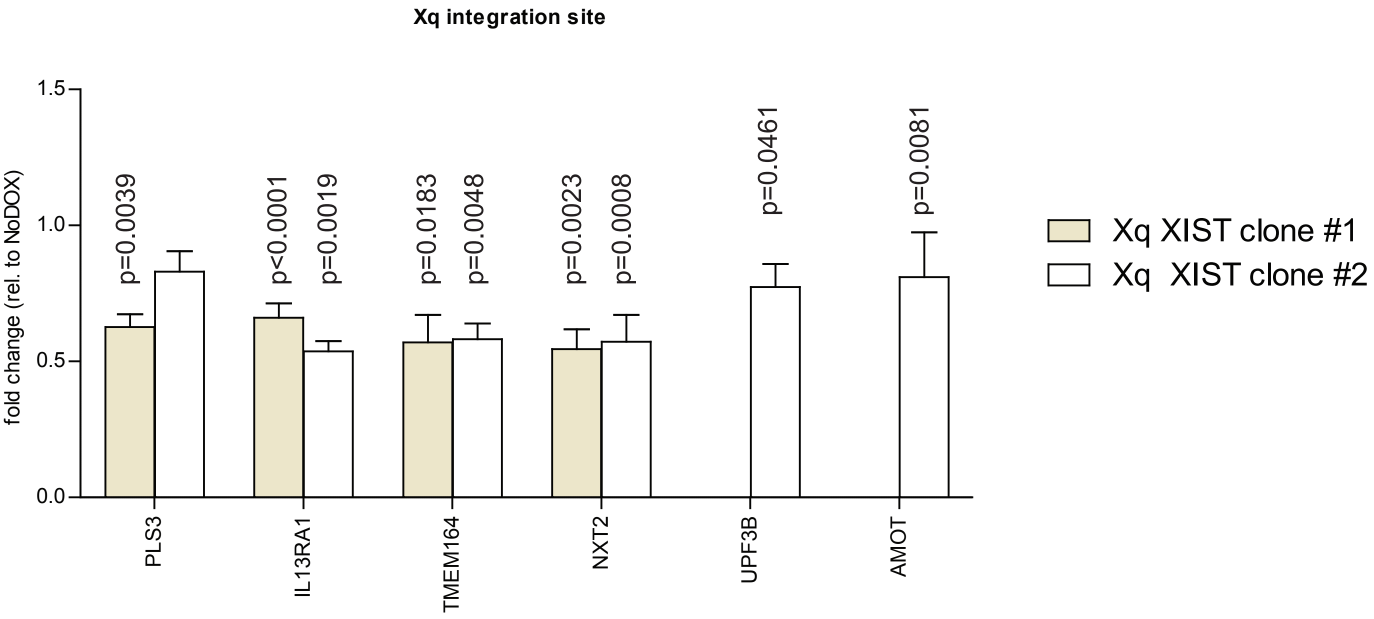

Xq integration site
fold change (rel. to NoDOX)
1.5
1.0
0.5
0.0
p=0.0039
p<0.0001
p=0.0019
p=0.0183
p=0.0048
p=0.0023
p=0.0008
p=0.0461
p=0.0081
PLS3
IL13RA1
TMEM164
NXT2
UPF3B
AMOT
Xq XIST clone #1
Xq XIST clone #2

c

1p

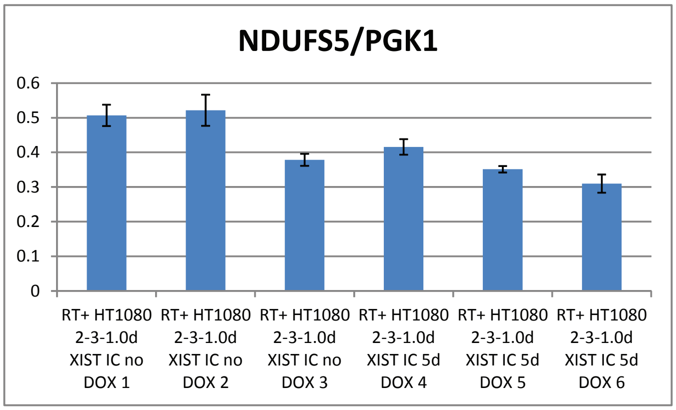
NDUFS5/PGK1
0.6
0.5
0.4
0.3
0.2
0.1
0
RT+ HT1080 2-3-1.0d XIST IC no DOX 1
RT+ HT1080 2-3-1.0d XIST IC no DOX 2
RT+ HT1080 2-3-1.0d XIST IC no DOX 3
RT+ HT1080 2-3-1.0d XIST IC 5d DOX 4
RT+ HT1080 2-3-1.0d XIST IC 5d DOX 5
RT+ HT1080 2-3-1.0d XIST IC 5d DOX 6

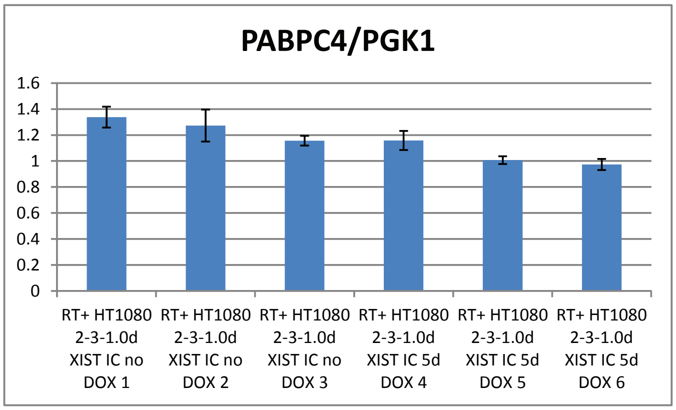
PABPC4/PGK1
1.6
1.4
1.2
1
0.8
0.6
0.4
0.2
0
RT+ HT1080 2-3-1.0d XIST IC no DOX 1
RT+ HT1080 2-3-1.0d XIST IC no DOX 2
RT+ HT1080 2-3-1.0d XIST IC no DOX 3
RT+ HT1080 2-3-1.0d XIST IC 5d DOX 4
RT+ HT1080 2-3-1.0d XIST IC 5d DOX 5
RT+ HT1080 2-3-1.0d XIST IC 5d DOX 6

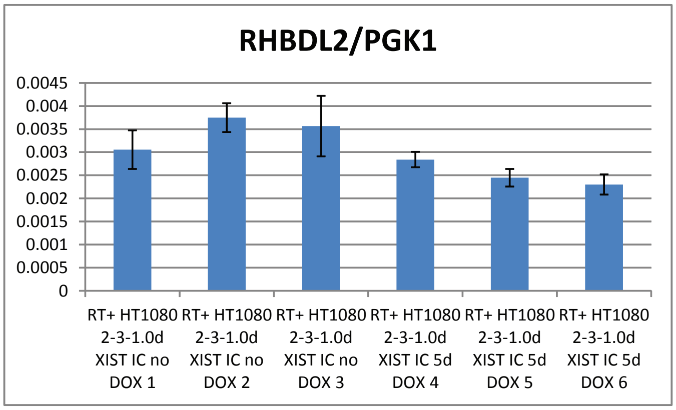
RHBDL2/PGK1
0.0045
0.004
0.0035
0.003
0.0025
0.002
0.0015
0.001
0.0005
0
RT+ HT1080 2-3-1.0d XIST IC no DOX 1
RT+ HT1080 2-3-1.0d XIST IC no DOX 2
RT+ HT1080 2-3-1.0d XIST IC no DOX 3
RT+ HT1080 2-3-1.0d XIST IC 5d DOX 4
RT+ HT1080 2-3-1.0d XIST IC 5d DOX 5
RT+ HT1080 2-3-1.0d XIST IC 5d DOX 6

7p

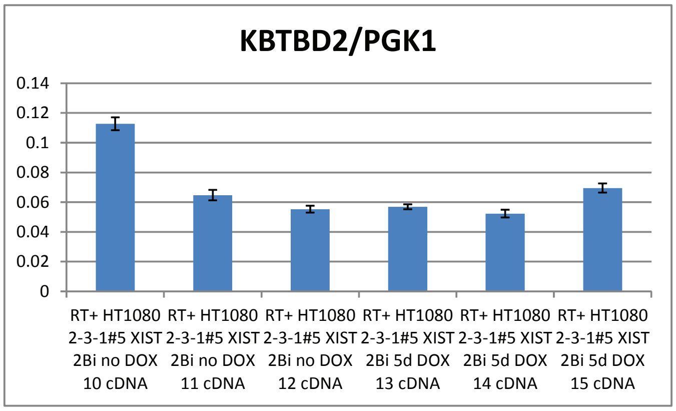
KBTBD2/PGK1
0.14
0.12
0.1
0.08
0.06
0.04
0.02
0
RT+ HT1080 2-3-1#5 XIST 2Bi no DOX 10 cDNA
RT+ HT1080 2-3-1#5 XIST 2Bi no DOX 11 cDNA
RT+ HT1080 2-3-1#5 XIST 2Bi no DOX 12 cDNA
RT+ HT1080 2-3-1#5 XIST 2Bi 5d DOX 13 cDNA
RT+ HT1080 2-3-1#5 XIST 2Bi 5d DOX 14 cDNA
RT+ HT1080 2-3-1#5 XIST 2Bi 5d DOX 15 cDNA

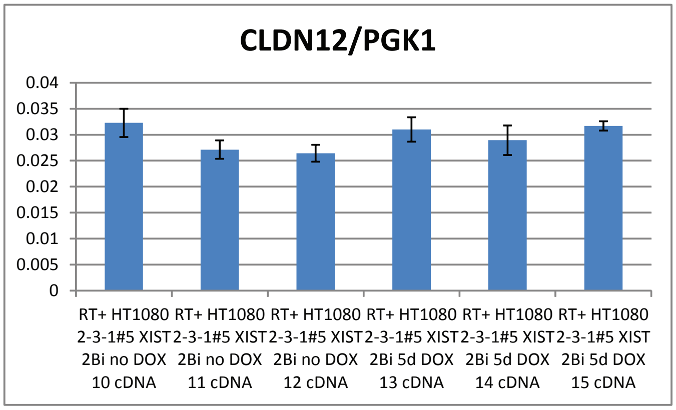
CLDN12/PGK1
0.04
0.035
0.03
0.025
0.02
0.015
0.01
0.005
0
RT+ HT1080 2-3-1#5 XIST 2Bi no DOX 10 cDNA
RT+ HT1080 2-3-1#5 XIST 2Bi no DOX 11 cDNA
RT+ HT1080 2-3-1#5 XIST 2Bi no DOX 12 cDNA
RT+ HT1080 2-3-1#5 XIST 2Bi 5d DOX 13 cDNA
RT+ HT1080 2-3-1#5 XIST 2Bi 5d DOX 14 cDNA
RT+ HT1080 2-3-1#5 XIST 2Bi 5d DOX 15 cDNA

7q

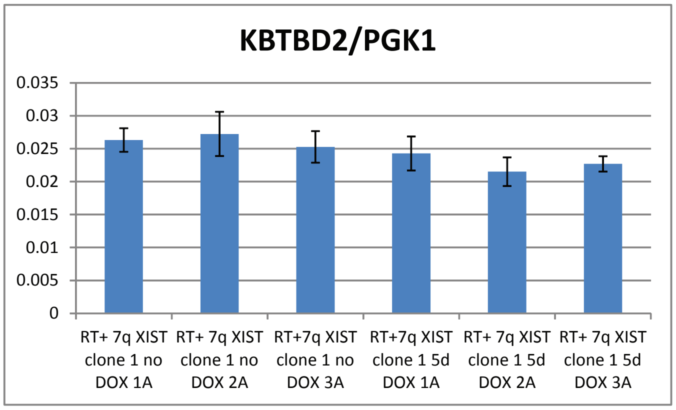
KBTBD2/PGK1
0.035
0.03
0.025
0.02
0.015
0.01
0.005
0
RT+ 7q XIST clone 1 no DOX 1A
RT+ 7q XIST clone 1 no DOX 2A
RT+7q XIST clone 1 no DOX 3A
RT+7q XIST clone 1 5d DOX 1A
RT+ 7q XIST clone 1 5d DOX 2A
RT+ 7q XIST clone 1 5d DOX 3A

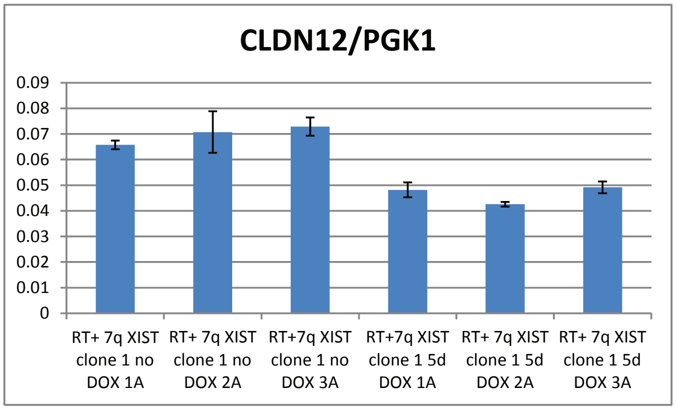
CLDN12/PGK1
0.09
0.08
0.07
0.06
0.05
0.04
0.03
0.02
0.01
0
RT+ 7q XIST clone 1 no DOX 1A
RT+ 7q XIST clone 1 no DOX 2A
RT+7q XIST clone 1 no DOX 3A
RT+7q XIST clone 1 5d DOX 1A
RT+ 7q XIST clone 1 5d DOX 2A
RT+ 7q XIST clone 1 5d DOX 3A

8p

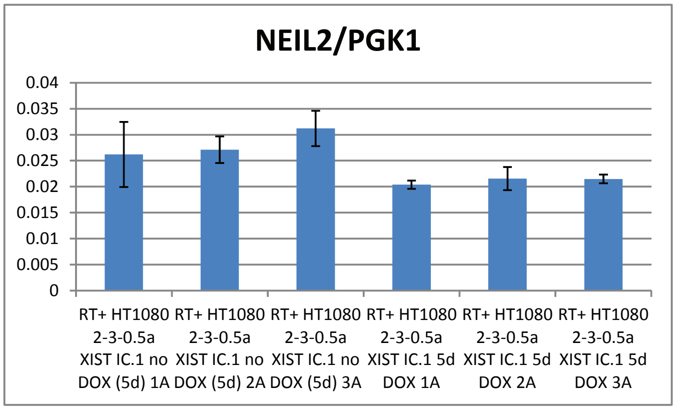

12q

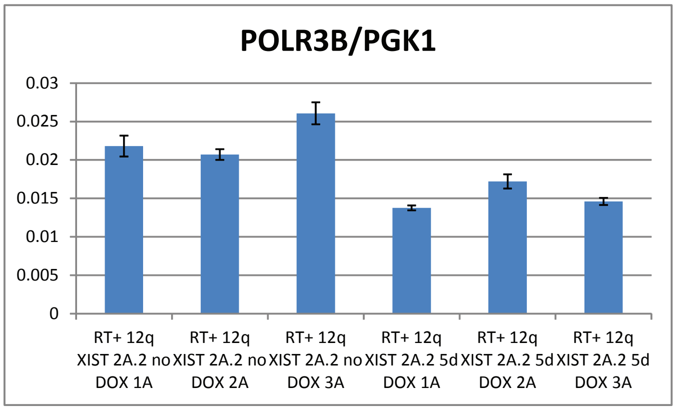

15q

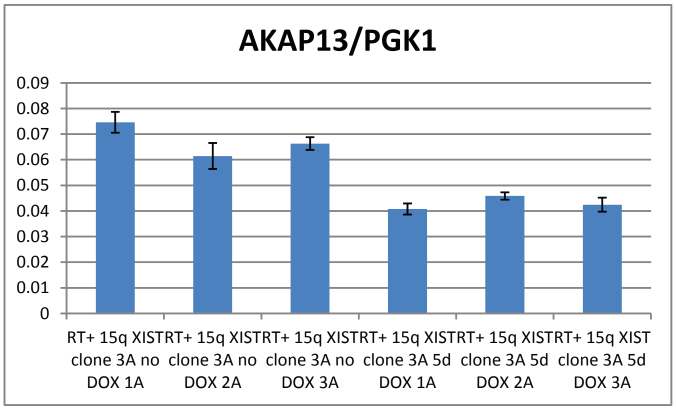

Additional Data File 3. Candidate gene silencing assays for each integration site.

a. Allelic pyrosequencing of three cDNAs from No DOX and DOX were compared in duplicate pyrosequencing reactions. As a control cDNA from a different integration was also assessed. One assay for each integration site was included in Figure 2.

b. For the X-chromosome integration site q-RT-PCR was used to determine silencing as the cells are hemizygous.

c. Q-RT-PCR assays were also performed to validate some autosomal silencing and compare with pyrosequencing and RNA-seq.
