# Supplementary material for: Impact of flanking chromosomal sequences on localization and silencing by the human non-coding RNA XIST
Source: Genome Biol. 2015 Oct 2;16:208. doi: 10.1186/s13059-015-0774-2 (PMC4591629; doi:10.1186/s13059-015-0774-2)
Supplement: Additional file 4: — Imprinted regions showed broad enrichment of H3K27me3 and punctate peaks of H3K27ac. Probes with values in the extreme 5 % of inputs were removed. (PDF 175 kb) [file 13059_2015_774_MOESM4_ESM.pdf]

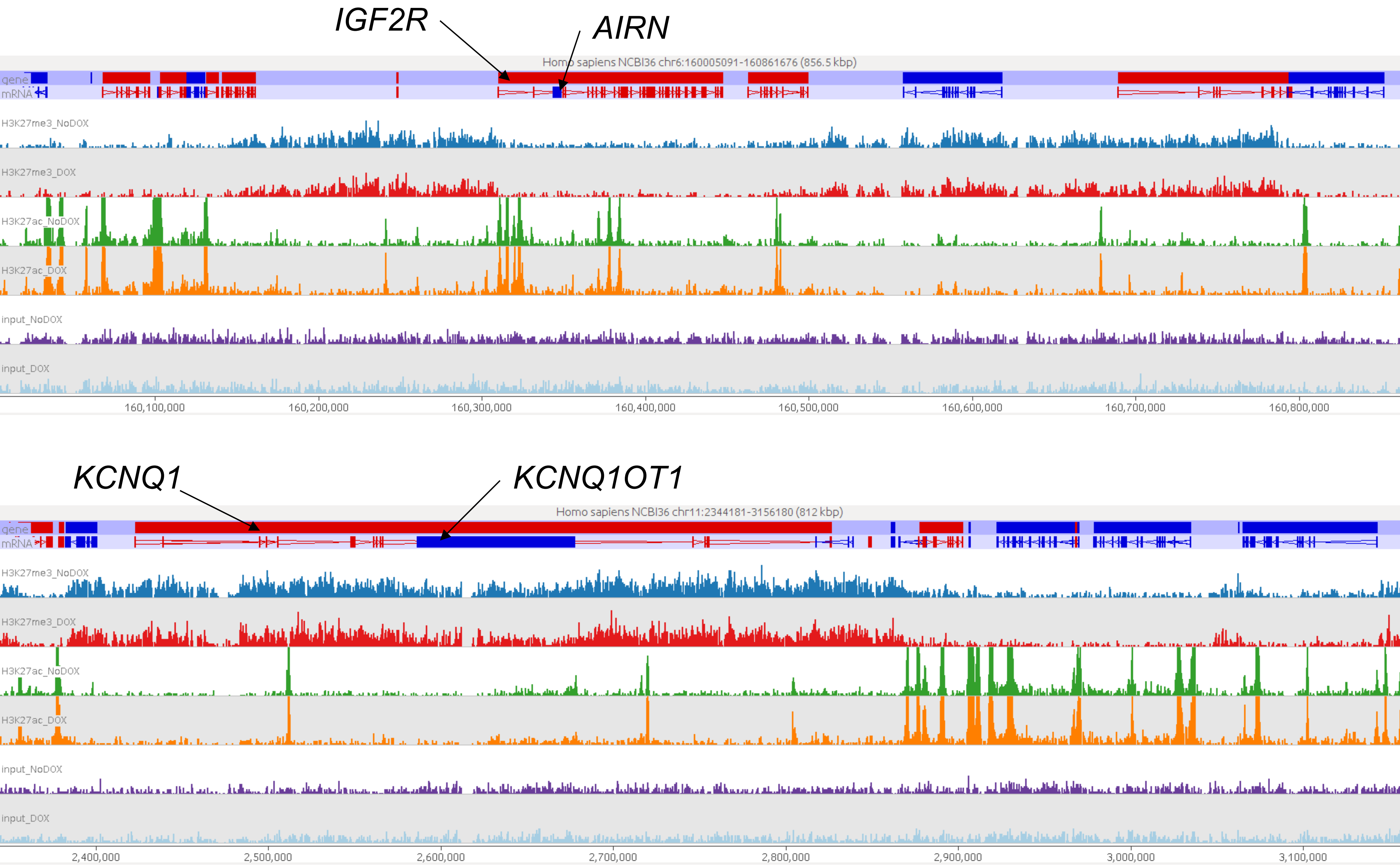

Additional Data File 4. Imprinted regions showed broad enrichment of H3K27me3 and punctate peaks of H3K27ac. Probes with values in the extreme 5% of inputs were removed.
