# Supplementary material for: Impact of flanking chromosomal sequences on localization and silencing by the human non-coding RNA XIST
Source: Genome Biol. 2015 Oct 2;16:208. doi: 10.1186/s13059-015-0774-2 (PMC4591629; doi:10.1186/s13059-015-0774-2)
Supplement: Additional file 5: — Average H3K27ac and H3K27me3 for genes on 8q and chromosome 1. The normalized ChIP-seq level are shown across an aggregate of genes and for the 10 kb upstream and downstream before (NoDOX) and after XIST expression (DOX). (PDF 246 kb) [file 13059_2015_774_MOESM5_ESM.pdf]

a

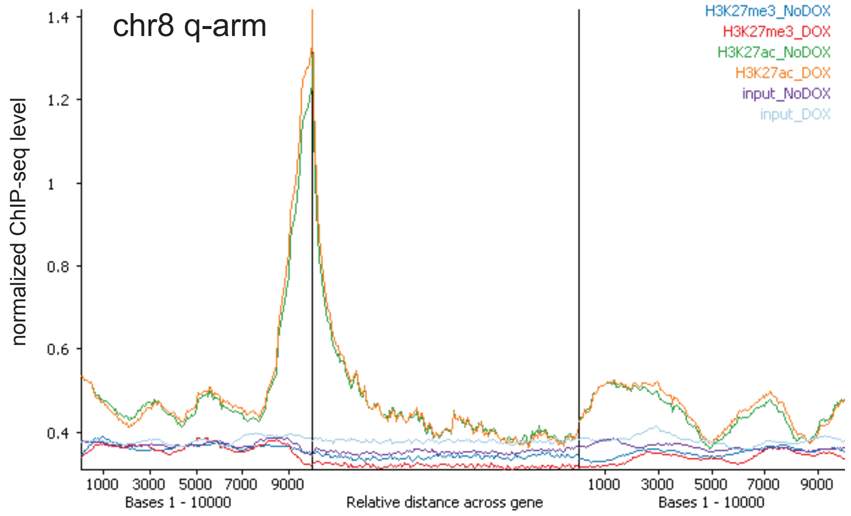

b

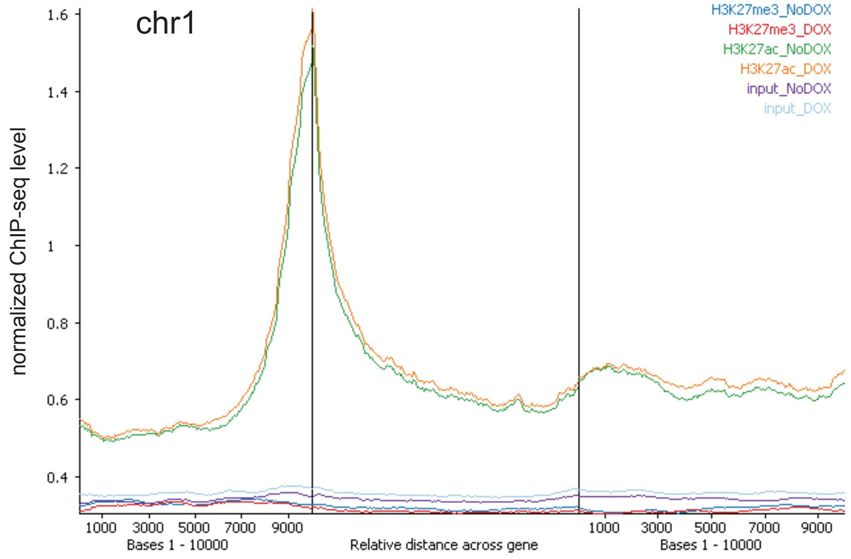

Additional Data File 5. Average H3K27ac and H3K27me3 for genes on 8q (a) and chromosome 1 (b). The normalized ChIP-seq level are shown across a normalized gene and for the 10 kb upstream and downstream before XIST expression (NoDOX) and after XIST expression (DOX).
